# Crimean-Congo Hemorrhagic Fever Virus Clades V and VI (Europe 1 and 2) in Ticks in Kosovo, 2012

**DOI:** 10.1371/journal.pntd.0003168

**Published:** 2014-09-25

**Authors:** Kurtesh Sherifi, Daniel Cadar, Skender Muji, Avni Robaj, Salih Ahmeti, Xhevat Jakupi, Petra Emmerich, Andreas Krüger

**Affiliations:** 1 Faculty of Agricultural and Veterinary Medicine, University of “Hasan Prishtina”, Prishtina, Kosovo; 2 Department of Virology, Bernhard Nocht Institute for Tropical Medicine, Hamburg, Germany; 3 Clinic of Infectious Diseases, University Clinical Centre, Prishtina, Kosova; 4 Department of Microbiology, National Institute for Public Health of Kosova, Prishtina, Kosova; 5 Department of Tropical Medicine, Bundeswehr Hospital Hamburg, Hamburg, Germany; University of California San Diego School of Medicine, United States of America

## Abstract

Despite being a small country, Kosovo represents one of the few foci of Crimean-Congo hemorrhagic fever (CCHF) in Europe. The distribution of Kosovar tick vectors and the evolution of CCHF virus in ticks are both as yet unknown. A better description of the extent and the genetic diversity of CCHFV in ticks from endemic settings is essential, in order to be controlled. We investigated the 2012 distribution of Kosovar ticks alongside the prevalence and the phylogeography of tick-derived CCHFV. *Hyalomma marginatum* dominated in the endemic municipalities with 90.2% versus 24.3% in the non-endemic regions. Of 1,102 tested ticks, 40 (3.6%) were CCHFV-positive, belonging to *H. marginatum* (29), *Rhipicephalus bursa* (10), and *Ixodes ricinus* (1). The virus strains clustered with clade V and VI related sequences. They fell into two lineages: Kosovo I and II. Kosovo I comprised strains recovered exclusively from *R. bursa* ticks and was closely related to AP92 prototype strain. Kosovo II clustered into Kosovo IIa, including human-derived strains, and IIb including only strains detected in *H. marginatum* and *I. ricinus*. Our phylogeographic reconstruction suggests two temporally distinct CCHFV introductions: the most probable location of the most recent common ancestor of Kosovo I lineage was in Greece (63 years ago) and that of lineages IIa-b in Turkey (35 years ago). After each CCHFV introduction into Kosovo, subsequent lineage expansions suggest periods of *in situ* evolution. The study provides the first insight into the genetic variability and the origin of CCHFV in ticks from Kosovo. Our findings indicate the spreading of CCHFV to non-endemic areas, which underlines the importance of further studies in order to monitor and predict future CCHF outbreaks in Kosovo. The AP92-like strains appear to be more widespread than previously thought and may provide a promising target for experimental studies due to their assumed low pathogenicity.

## Author Summary

Crimean-Congo hemorrhagic fever virus (CCHFV) is the most widespread tick-borne virus affecting humans. Its distribution coincides with that of Old World *Hyalomma* tick vectors. The virus circulates in an enzootic cycle, where hard ticks are both vectors and reservoirs, and mammals the amplifying hosts. Accidentally, humans become infected as dead-end hosts. One of the few CCHF-endemic European countries is Kosovo, where an annual average of ten sporadic cases, with a mean mortality rate of 18%, are reported. In this study, the tick distribution, CCHFV prevalence and the phylogeography of the tick-derived strains from 2012 were investigated. The majority of ticks belonged to *Hyalomma marginatum*, the main vector of CCHFV. Indeed, of 40 CCHFV-positive ticks, 29 were *H. marginatum*. The phylogenetic analyses revealed that 30 viral strains formed a distinct lineage within clade V (Europe 1) and were closely related to previously known strains from humans and ticks from the Balkans. However, ten isolates from *Rhipicephalus bursa* ticks, collected in a non-endemic region, turned out to cluster within a very divergent CCHFV clade (Europe 2), so far only known from Greece and Turkey. Since these strains presumably develop only subclinical or mild infections in humans, they might be suitable for experimental vaccine studies.

## Introduction

Crimean-Congo hemorrhagic fever virus (CCHFV) is the most widespread tick-borne arbovirus (Bunyaviridae: *Nairovirus*) with an Old World distribution ranging from southern and western Africa via the Mediterranean basin to the Middle East and China (Ergonul & Whitehouse 2007). The virus circulates in an enzootic cycle, whereby ticks (Acari: Ixodidae) are both vectors and reservoirs, while mammals are the amplifying hosts. The main vectors are species of the genus *Hyalomma*, and it is evident that the distribution of the virus correlates to that of the *Hyalomma* vectors [1]. In the endemic areas, CCHFV is sporadically transmitted to humans via tick bites or by contact with viremic animals or humans. Nosocomial transmission has also been reported. Human infections show a rather high case-fatality rate of up to 80% [1], [2]. Persons at risk are most frequently found amongst farmers and their families, as well as slaughterhouse and healthcare workers, veterinarians, or military personnel [3]. However, compared with other arboviruses, CCHFV displays a rather high degree of genetic diversity leading to seven phylogeographic clusters [4]. Furthermore, the different strains are probably correlated with varying degrees of pathogenicity [3]. One of the few CCHF-endemic countries known in Europe is Kosovo, from where the disease was first reported in 1954 [5], [6]. At least since 1995, human cases occur every year, with an average mortality rate of 25.5% [7]. In 2012, the National Institute of Public Health of Kosovo confirmed 13 cases by PCR, which is approximately the average number of annual cases between 1999 and 2009. Most cases originated in the central parts of the country, in particular the Malishevë municipality, which is considered one of the hyper-endemic regions of Kosovo [6]. Until now little was known about the vector populations and virus prevalence in ticks from Kosovo. According to various sources, there are up to 29 ixodid species in former Yugoslavia [8]. *Hyalomma marginatum* (sensu [9], syn. *H. plumbeum plumbeum*), the main vector in the Mediterranean region, was only reported from Kosovo since 1967 [10]. By then the species already accounted for about half of the total tick population on domestic animals, along with the less abundant *H. anatolicum*. Previous workers had used misleading tick species names, e.g. *H. scupense*, *H. detritum*, *H. aegyptium* [11], which were applied by other workers to other species. In fact, the taxonomy of the genus *Hyalomma* is still not fully understood [9], [12], and therefore it is impossible to compare the historic records with the present situation. Regarding CCHFV prevalence in ticks from former Yugoslavia, only few data are available. Gligic et al. [13] isolated two strains from 10 pools of *H. marginatum* and one strain from an *Ixodes ricinus* pool, all collected in 1973 near Tetovo in northern Macedonia (former Yugoslav Republic of Macedonia), following a family outbreak of CCHF in 1970. Out of 273 *H. marginatum* taken from cattle following the 2001 outbreak, 41 (15%) tested CCHFV-positive by PCR when examined by Duh et al [14]. The present study aims to establish a baseline of (i) the current tick fauna and distribution, (ii) the CCHFV prevalence in ticks from Kosovo, using real-time RT-RCR, and (iii) the phylogenetic and phylogeographic relationship of the tick-derived CCHFV strains.

## Methods

### Study area

**Figure 1:**
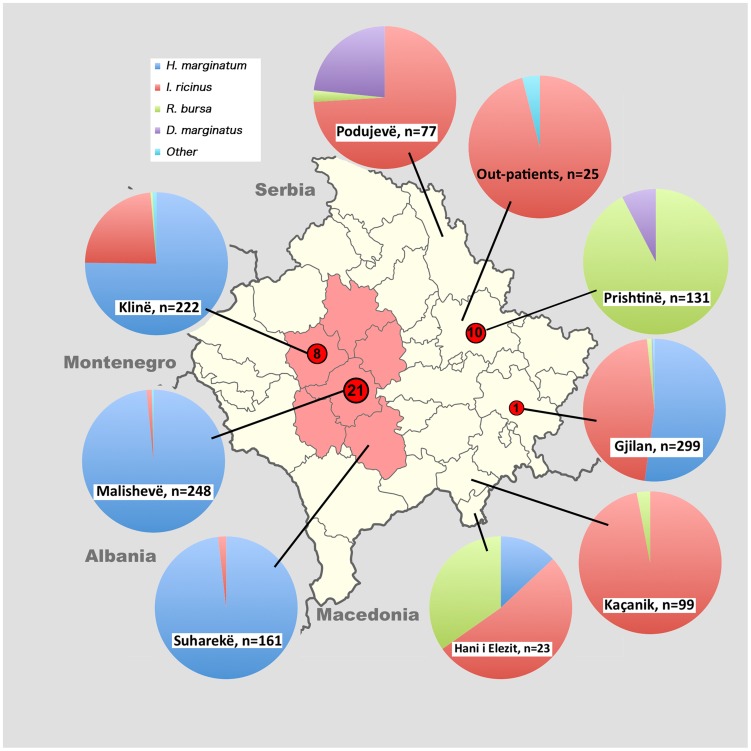
Ixodid tick species distribution in Kosovo, 2012. Also shown are the municipality borders. In red are the CCHF-hyper-endemic municipalities according to Jameson et al. (2012). The numbers in the red circles indicate virus-positive tick samples.

With a total area of 10.887 km^2^ Kosovo is the smallest of the ex-Yugoslav republics. It is a land-locked, mountainous country at elevations between 276 and 2.656 meters a.s.l., whereby the lower central basins are surrounded by high mountain ranges. The climate is continental. According to Jameson et al. [6], the CCHF risk areas cover approximately 50% of municipalities, with hyper-endemicity being found in Skënderaj, Klinë, Malishevë, Rahovec and Suharekë municipalities (Fig. 1). The prevailing CCHF season is June and July. In order to survey a broad geographical spectrum, ticks were collected in villages of eight of the 38 municipalities, namely the CCHF-endemic municipalities Malishevë (three villages), Klinë (six villages) and Suharekë (five villages), and the non-endemic municipalities Gjilan (11 villages), Podujevë (five villages), Prishtinë (one village), Hani i Elezit (one village) and Kaçanik (three villages).

### Tick collections

Where possible, ticks were collected from livestock, i.e. goats, cattle and sheep, or in the animals' pastures. This was mainly achieved in the endemic villages. If no livestock was present, e.g. at higher altitudes in the peripheral municipalities Kaçanik and Hani i Elezit, ticks were collected by flagging or directly from the ground surface. In addition, a limited sample from human bait was obtained from out-patients attending the Infectious Diseases Department of Prishtinë University hospital. All samples were collected during May and early June 2012. During all fieldwork, all participants used disposable personal protective equipment, i.e. latex gloves, coverall suit, goggle and face mask. Subsequent to removing them from animals with fine forceps' or picking them up from the ground, ticks were put into 2 ml cryovials, and transferred to a −80°C freezer in the veterinary lab. First-hand morphological identifications and sex determination (only adult ticks were collected) were conducted according to Estrada-Peña et al. [15], using a chill-table and a dissection microscope. Specimens with uncertain species identity were excluded from subsequent virus screening, but were further identified using a reference collection in the Bernhard Nocht Institute for Tropical Medicine (BNI) and specialized keys [9], [16], and kept as morphological vouchers. The molecular species identity of *H. marginatum* from Kosovo had been previously confirmed by *COI* barcoding (A. Krüger, unpublished).

### RT-PCR detection of CCHFV

After transport into a BSL-3 facility equipped with a glove box, single ticks were transferred into 2 ml Eppendorf tubes with two 7 mm steal beads, frozen on liquid nitrogen and immediately pulverized in a Tissue Lyser LT (Quiagen, Hilden, Germany) with a −20°C pre-cooled rotor at 50 Hertz for 2 to 12 minutes. In some cases, repeated freezing was necessary. The samples were then resuspended in 300–500 µl PBS (depending on tick size) containing 10% fetal calf serum, 500 IU/ml penicillin and 500 µg/ml amphothericin, and centrifuged at 2000 rpm. For viral RNA extraction, either 140 µl of the supernatant was mixed with 560 µl AVL buffer (QiaAmp Viral RNA Minikit, Qiagen, Hilden, Germany) and lysed at room temperature for 10 min, or 200 µl of the supernatant was diluted in 200 µl distilled water including carrier RNA and proteinase K (RTP DNA/RNA Virus Mini Kit, Stratec, Birkenfeld, Germany). We then followed the instructions of the kit's manufacturers. For first round diagnostic RT real-time PCR, five µl RNA extractions (each of up to six single tick extractions) were assembled into 218 pools. Single tick diagnostic PCRs were subsequently conducted for CCHFV-positive pools alone. The *Hyalomma marginatum* pool PCRs were carried out using both the AgPath-ID One-Step RT-PCR kit (Life Technologies, Carlsberg, CA), modified after Atkinson et al. [17], and the RealStar CCHFV RT-PCR Kit 1.0 (Altona Diagnostics, Hamburg, Germany), according to the manufacturers' instructions. Modifications of the former were as follows: modified primers CCHF S1-flap 5-AATAAATCATAATCTCAAAGAAACACGTGCC-3′ and CCHF S122-flap 5′-AATAAATCATAACCTTTTTGAACTCTTCAAACC-3′. All non-*Hyalomma* pool PCRs and all single tick PCRs from positive pools were conducted with the RealStar CCHFV RT-PCR Kit only. PCRs were run and analyzed on a Rotor-Gen 6000 platform (Corbett Research, Mortlake, Australia).

### cDNA synthesis and amplification of capsid protein gene (S segment) fragments

In order to further characterize the virus strains and to compare them with previously published data, a 293 bp length fragment of the nucleoprotein (S segment) gene was amplified by RT-PCR using designed degenerate forward primer CCHFVD-F (5′-AAGTTCTGTGCACCHATATATG-3′) at position 269 and reverse primer CCHFVD-R (5′-CATTTCTTTRACAGRCAYCA-3′) at position 562, both positions according to the full capsid protein gene sequence of the Hoti strain (GenBank acc. no. DQ133507). Reverse transcription and PCR amplification (RT-PCR) of the 293 bp fragment of the nucleoprotein gene was performed using the SuperScript III One-Step RT-PCR System with Platinum Taq High Fidelity (Invitrogen, Carlsbad, CA). The PCR was performed in a 50 µl volume containing 3 µl of sample RNA, 25 µl of 2× reaction mix, 2.5 µl of each primer, 1 µl High Fidelity Enzyme mix, and ddH_2_O up to 50 µl. Amplification conditions were as follows: reverse transcription was performed at 50°C for 50 minutes, followed by denaturation at 94°C for 2 min.; 45 amplification cycles were performed at 94°C for 20 s, 55°C for 45 s, 72°C for 1 min. Final extension was at 72°C for 7 min. The fragments were sequenced from both ends by conventional Sangers technology by LGC Company (Berlin, Germany).

### Nucleotide sequence data set

The data set (n = 577) comprised 40 Kosovar sequences derived during the study, together with 16 previously reported in Kosovo and all corresponding CCHFV partial S segments available in the databases. Only Genbank submissions that included both a date and location of origin were considered. Table S1 shows details of all sequences used in the study including accession numbers, dates, species and countries of origin. Alignment was performed using the MAFFT algorithm, then visually inspected using Geneious R7 v7.1.4, created by Biomatters (available from http://www.geneious.com). In order to avoid overloading of the data set, identical sequences (duplicates) from the same year of isolation and country of origin were excluded. This reduced the original set of 577 sequences to a subset of 270 sequences that provided the greatest coverage of geographical regions and time of collection. Recombination events complicate the use of phylogenetic parameters such as timing events and selection pressures [18]. Considering reports of recombination events found in S segments [19], the nucleotide sequence alignment was screened for recombination using RDP [20], GENECONV [21], Chimaera [22], MaxChi [23] and Bootscan [24] methods implemented in RDP4 [25]. In our dataset alignment, no consensus for recombination events was found by applying the aforementioned methods.

### Construction of time-scaled phylogenies and phylogeographic analyses

In order to investigate the evolutionary relationships of the Kosovar CCHFV strains with those previously reported worldwide, we performed a Bayesian Monte Carlo Markov Chain (MCMC) sampling method implemented in BEAST v1.7.5 package [26], which estimates the substitution rates, divergence times and demographic histories of the sampled lineages. The best nucleotide substitution model detected by the Akaike Information Criterion (AIC) in jModelTest 2 [27] was found to be the General-Time-Reversible model of sequence evolution with gamma-distributed rate variation among sites and a proportion of invariable sites (GTR+Γ+I). A relaxed uncorrelated lognormal clock and the Bayesian skyline plot (BSP) coalescent model [28] with 20 coalescent-interval groups were found to best fit the data. To test the hypothesis that CCHFV is periodically introduced into Kosovo from the neighbouring countries, a phylogeographic analysis was conducted, using a reversible discrete diffusion model also implemented in BEAST [29]. This diffusion model uses the countries of the sampled isolates to reconstruct the ancestral location states of the internal nodes from the posterior time-scaled tree distribution. Two independent runs of 100 million generations sampled every 10,000 steps were performed to estimate the posterior probability distribution. The first 10% steps of each run (such that ESS values were >200) were discarded as burn-in and to ensure that convergence was assessed we used TRACER program (http://tree.bio.ed.ac.uk/software/tracer/). To obtain the maximum credibility consensus tree the softwares TreeAnnotator v1.5.4 and FigTree v1.3.1 (http://tree.bio.ed.ac.uk/software/figtree/) were implemented.

### Accession numbers

The nucleotide sequences generated in this study have been deposited in GenBank under accession numbers KJ545665 – KF545704.

## Results

### Tick species of Kosovo

A total of 1285 ixodid ticks were collected in eight municipalities of Kosovo in May and June 2012 (Table 1). The ticks belonged to seven species, namely *Hyalomma marginatum* (56.7%), *Ixodes ricinus* (30%), *Rhipicephalus bursa* (10.7%), *Dermacentor marginatus* (2.3%), *Rhipicephalus* (*Boophilus*) *annulatus* (0.2%), *Haemaphysalis punctata* (0.2%) and *Haemaphysalis inermis* (0.1%). If analysed separately according to the pre-defined CCHF endemicity, *H. marginatum* dominated in the endemic municipalities with 90.2% as opposed to 24.3% in the non-endemic regions (Fig. 1). In the non-endemic regions, however, *I. ricinus* (50%) and *R. bursa* (20.9%) were the most abundant species, with the exception of Gjilan municipality, where *H. marginatum* accounted for the majority (52.2%).

**Table 1:** Number and distribution of tick species in Kosovo, 2012.

		No. of specimens collected per species no. (% of total region)							
Region	Municipality	*Hyalomma marginatum*	*Ixodes ricinus*	*Rhipicepha-lus bursa*	*Dermacentor marginatus*	*Rhipicephalus* (*B.*) *annulatus*	*Haemaphysa-lis punctata*	*Haemaphysa-lis inermis*	TOTAL
Endemic	Malishevë	244	3			1			248
	Klinë	167	52	1			1	1	222
	Suharekë	158	3						161
	Sub-TOTAL	569 (90.2%)	58 (9.2%)	1 (0.2%)	0	1 (0.2%)	1 (0.2%)	1 (0.2%)	631
Non-endemic	Podujevë		57	2	18				77
	Prishtinë			121	10				131
	Gjilan	156	138	3	1	1			299
	Kaçanik		96	3					99
	Hani i Elezit	3	12	8					23
	Hospital		24				1		25
	Sub-TOTAL	159 (24.3%)	327 (50%)	137 (20.9%)	29 (4.4%)	1 (0.2%)	1 (0.2%)	0	654
	**TOTAL**	**728 (56.7%)**	**385 (30%)**	**138 (10.7%)**	**29 (2.3%)**	**2 (0.2%)**	**2 (0.2%)**	**1 (0.1%)**	**1285**

### CCHFV prevalence in the tick populations

CCHF viral RNA could only be detected in ticks from four out of the eight investigated municipalities (Fig. 1). Of the 1102 tested ticks (85.8% of all collected ticks), 40 (3.6%) were tested virus-positive, belonging to *H. marginatum*, *R. bursa* and *I. ricinus* (Table 2). Most positive ticks were found in Malishevë and Klinë municipalities (29 ticks), which belong to the hyper-endemic regions anyway. Here, 28 positive *H. marginatum* and one positive *I. ricinus* were detected, whereby the positivity rate of *H. marginatum* per village ranged from 1.25% to 29.4% (average 11%). However, in several other villages of Klinë municipality and all the villages in Suharekë municipality no positive ticks were detected. The remaining 11 positive ticks were found in regions previously regarded as non-endemic. In a rural outskirt of Prishtinë, 10 out of 108 (9.3%) *R. bursa* ticks were virus-positive. In Gjilan one out of 30 (3.3%) *H. marginatum* was positive. In order to standardize the regional differences in the total number of ticks against virus positivity, the following minimum infection rates (MIR; [number of positive specimens/total specimens tested]×1000) were calculated. For (i) positive regions: Malishevë = 86.4, Klinë = 40.4, Prishtinë = 84.7, Gjilan = 4.98. For (ii) negative regions, one can only provide a theoretical MIR, assuming that one more specimen from each region (total+1) could have been positive, as follows: Suharekë = 6.2, Podujevë = 15.6, Hani i Elezit and Kaçanik = 8.4. The MIRs reflect much higher virus prevalence (>40 positive ticks per 1000) in the endemic regions Malishevë and Klinë as well as Prishtinë, whereas Gjilan, a previously non-endemic region, resembles the situation in the presently negative regions (<16 per 1000).

**Table 2:** Number and origin of ticks tested CCHFV-positive by RT real-time PCR in Kosovo, 2012.

					Prevalence per species (%)		
Region Municipality	Village	Collected from	No. of tested ticks	No. of CCHFV positive (%)	*H. marginatum* (%)	*R. bursa*	*I. ricinus*
**Endemic**							
Malishevë	Bubavec	Goat	80	1 (1.25%)	1/80 (1.25%)		
	Bubël	Cow	68	20 (29.4%)	20/68 (29.4%)		
	Vërmnicë	Goat	95	0			
		**Sub-TOTAL**	243	**21 (8.6%)**	**21/148 (14.2%)**		
Klinë	n.d.	n.d.	91	4 (4.4%)	3/84 (3.6%)		1/7
	Rixhevë	Animal	18	1 (5.6%)	1/12 (8.3%)		
	Përqevë	Animal	26	3 (11.5%)	3/23 (13%)		
	4 villages^1^	Animal, Ground	66	0			
		**Sub-TOTAL**	198	**8 (4.0%)**	**7/119 (5.9%)**		**1/7 (14.3%)**
Suharekë	5 villages^2^	Cow, ground	161	0			
**Non-endemic**							
Podujevë	5 villages^3^	Cow	63	0			
Prishtinë	Hajvali	Sheep	118	**10 (8.5%)**		**10/108 (9.3%)**	
Gjilan	4 villages^4^	Cow	157	0			
	Gjilan	Cow	44	1 (2.3)	1/30		
		**Sub-TOTAL**	201	**1 (0.5)**	**1/30 (3.3%)**		
Kaçanik+Hani i Elezit	4 villages^5^	Ground	118	0			
**TOTAL for all regions**			**1102**	**40 (3.6%)**	**29/297 (9.8%)**	**10/108 (9.3%)**	**1/7 (14.3%)**

1 Cerovike, Terdevcë, Gjurgjevik i M., Rhixere;

2 Nishor, Kasterrce, Doberdelan, Semetishte, Samadraxhe;

3 Podujeve, Badovce, Orllate, Prapashtice, Lupc;

4 Llashtic, Perlepnicë, Sllakovc, Muçibabë;

5 Gerlice e Eperme, Gabrice, Bajnice, Paldenice.

### Phylogenetic analysis of Kosovar CCHFV strains from ticks

**Figure 2:**
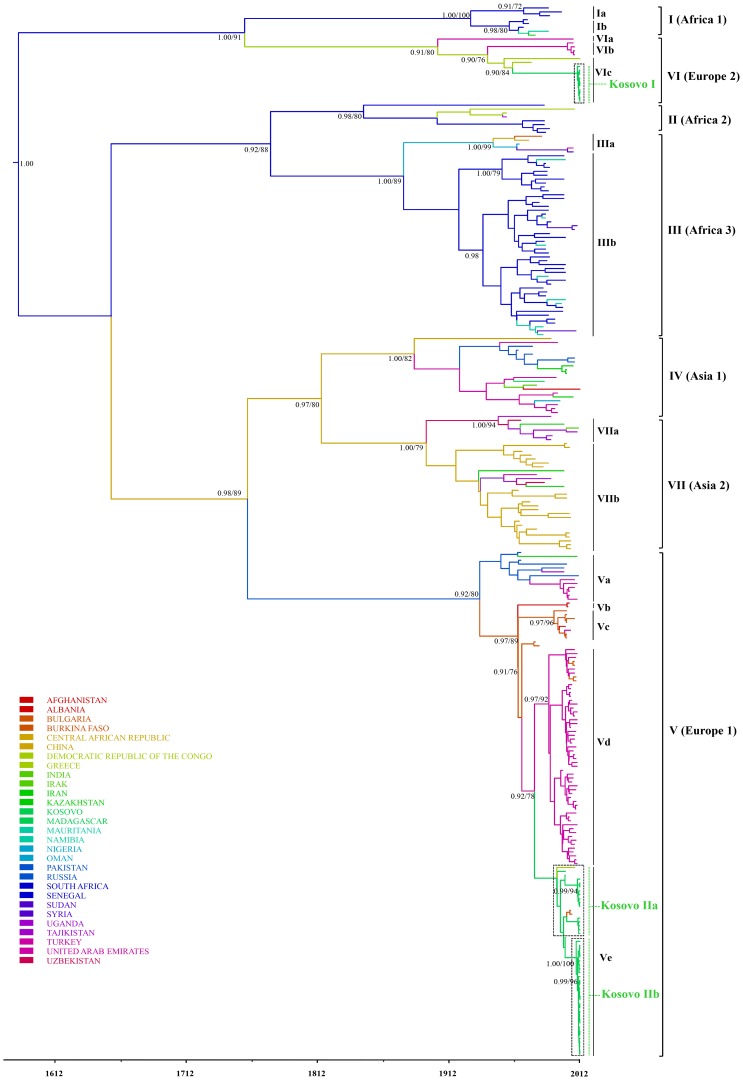
Bayesian maximum clade credibility (MCC) tree representing the time-scale phylogeny of CCHFV, as obtained by analysis of 293 long genomic fragments of the nucleoprotein (S segment) coding sequences. The branches are colored on the basis of the most probable location state of the descendent nodes (see color codes). Numbers at the nodes indicate posterior probability values (clade credibilities ≥90%) and parallel maximum likelihood bootstrap replicates (≥70%). The scale at the bottom of the tree represents the years before the last sampling time (2012). The main clades (genotypes), subclades and Kosovar lineages (dotted squares) are indicated to the right of the tree. The human and tick-derived CCFHV strains are also indicated. Tick-derived sequences derived from this study are designated with “Kosovo I” and “Kosovo IIb”. All remaining sequences were extracted from public databases.

**Figure 3:**
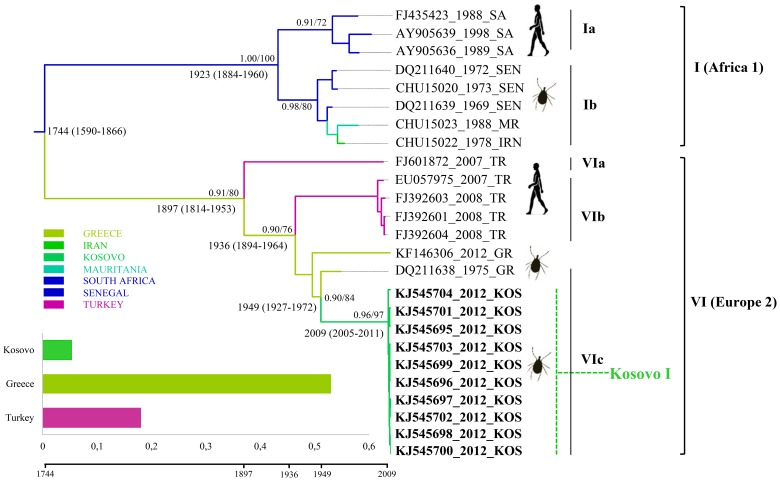
Enlargement of the section of MCC tree containing Kosovar lineage I. The estimated dates of divergence from neighbouring sister lineages and mean dates of existence for the most recent common ancestor (MRCA) for lineage containing Kosovar sequences (with 95% HPD in parentheses) are shown at the relevant nodes. The branches are coloured on the basis of the most probable location of the descendent nodes (GR, Greece; IRN, Iran; KOS, Kosovo; MR, Mauritania; SA, South Africa; SEN, Senegal; TR, Turkey). Histogram insertions indicate the location state probabilities for the estimated introductions of CCHFV that gave rise to Kosovo lineage I. Taxon labels include accession number, year of isolation, and country of origin. The human and tick-derived CCFHV strains are also indicated. Tick-derived sequences generated in this study are bolded (clade “Kosovo I”).

**Figure 4:**
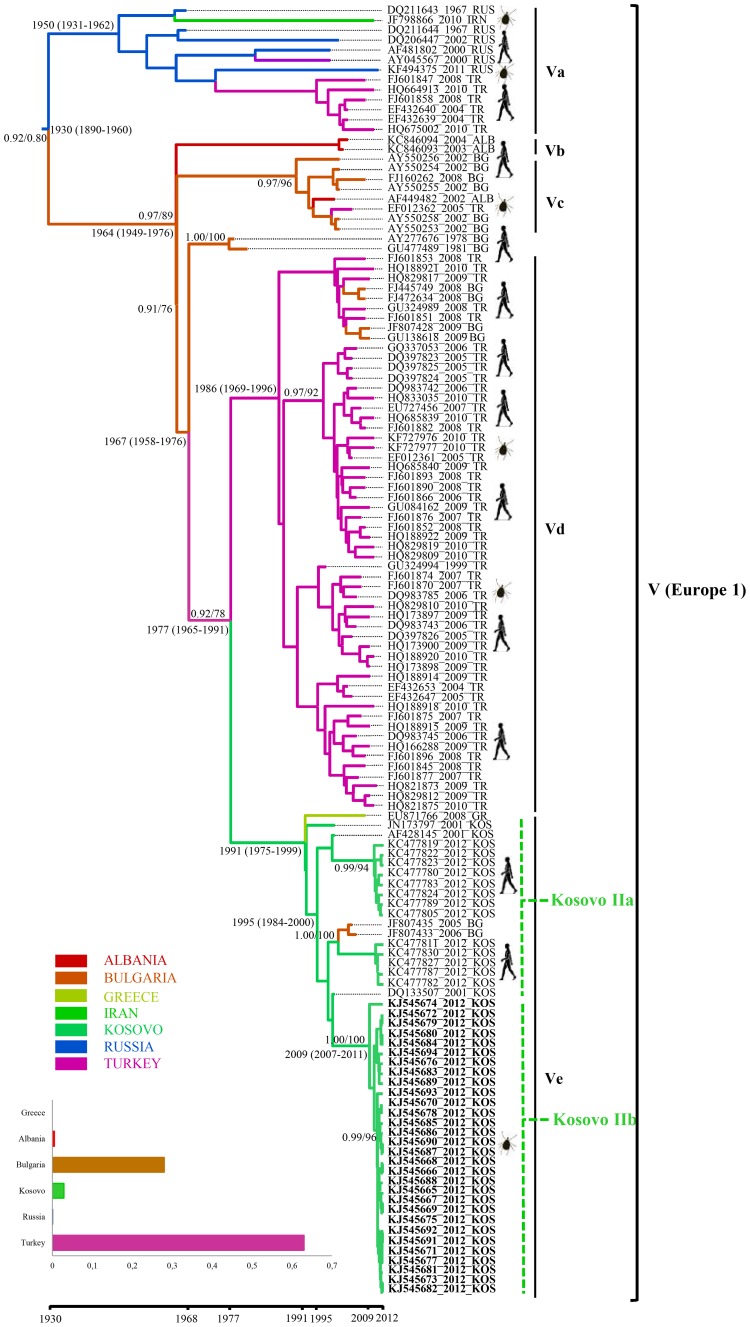
Enlargement of the section of MCC tree containing Kosovar lineages IIa-b. The estimated dates of divergence from neighbouring sister lineages and mean dates of existence for the most recent common ancestor (MRCA) for lineages containing Kosovar sequences (with 95% HPD in parentheses) are shown at the relevant nodes. The branches are coloured on the basis of the most probable location of the descendent nodes (ALB, Albania; BG, Bulgaria; GR, Greece; IRN, Iran; KOS, Kosovo; RUS, Russia; TR, Turkey). Histogram insertions indicate the location state probabilities for the estimated introductions of CCHFV that gave rise to Kosovo lineages IIa-b. Taxon labels include accession number, year of isolation, and country of origin. The human and tick-derived CCFHV strains are also indicated. Tick-derived sequences generated in this study are bolded (clades “Kosovo IIa-b”).

All PCR-confirmed CCHFV-positive samples from Kosovar ticks were processed for partial S segment sequencing, adding 40 new CCHFV sequences to the publicly available data set. The phylogenetic relationship was inferred from 230 previously published CCHFV strains and 40 new sequences (Figs. 2–4). The Bayesian maximum clade credibility (MCC) tree inferred from the partial data set of CCHFV S segment sequences is divided into seven major clades (Fig. 2). All of the seven main clades corresponding to the viral genotypes were confirmed by the maximum likelihood inference and supported by bootstrap values (≥70). The phylogenetic analyses show that all the strains from *H. marginatum* and *I. ricinus* in this study, together with previously reported Kosovar CCHFV strains from humans, formed two distinct lineages in clade V (Europe 1) of CCHFV viruses (Figs. 2, 4), whereas the remaining 10 from *R. bursa* fell in clade VI (Europe 2), also forming a distinct lineage within this clade (Fig. 3). Clade VI (Europe 2), which comprises of CCHFV strains/isolates associated with humans and ticks, clusters into three well-supported lineages (posterior probability ≥0.90) from which subclades VIa-b encompass human derived sequences from Turkey, while subclade VIc contains only tick-associated strains from Greece (AP92 strain) and Kosovo. Here the Kosovar tick-derived CCHFV strains fell into a distinct and well-supported lineage named Kosovo I (Figs. 2, 3). Within clade V (Europe 1) there are five well-defined subclades designated as Va-Ve. Subclade Va contains ticks and human-derived CCHFV sequences from Russia, Turkey and Iran, subclade Vb contains human strains exclusively from Albania, while subclade Vd has its origins in both Turkey and Bulgaria (Figs. 2, 4). Subclade Ve comprises sequences originating from Kosovo (except one strain from Greece and two from Bulgaria), where the tick and human-derived CCHFV strains clustered into two distinct lineages (Kosovo IIa and IIb) (Figs. 2, 4). The nucleotide sequence alignment of 30 subclade Ve strains generated from ticks during this study with those previously isolated from humans from Kosovo showed sequence similarities ranging from 96.3% to 98%. In terms of the spatial distribution of the tick-derived CCHFV sequences, 29 strains belonging to clade V originated from the endemic municipalities and only one from a non-endemic region (Gjilan). In contrast, all 10 strains from clade VI were found in a non-endemic area (Prishtinë).

### Inference of evolutionary rates, dates of divergence and geographic origins of Kosovar CCHFV

The variations of CCHFV genome sequences combined with sample collection dates and locations can help to identify the source and the evolutionary history of the new strains. The phylogeographic analysis suggests two distinct introductions of CCHFV into Kosovo from neighboring countries (Figs. 3, 4). Kosovo lineage I is thought to be a descendant of CCHFV that most probably existed in Greece (state posterior probability = 0.54) around 1949 (95% HPD [high posterior density] 1927–1972). In the case of Kosovo lineages IIa and IIb the most recent common ancestor (MRCA) is estimated to have descended from an ancestor that existed in Turkey around 1977 (95% HPD 1965–1991; state posterior probabilities probability = 0.62). Lineage expansion after tick/human-derived CCHFV introduction is estimated to have occurred within Kosovo around 2009 (95% HPD 2005–2011) in the case of lineage I, around 1991 (95% HPD 1975–1999) for lineage IIa, and 2009 (95% HPD 2007–2011) for lineage IIb. One critical feature of emerging viruses is how quickly they change. The overall mean rate of evolution estimated for all tick and human-derived CCHFV sequences for clade V is 1.71×10^−4^ (95% confidence interval [CI],1.25×10^−4^–2.30×10^−4^) subs site^−1^ year^−1^ and 2.45×10^−3^ (95% confidence interval [CI], 1.95×10^−5^–4.14×10^−4^) for clade VI compared with 5.71×10^−4^ (95% confidence interval [CI], 3.98×10^−4^–6.66×10^−4^) subs site^−1^ year^−1^ across all of the sampled CCHFV variant lineages. The estimated mean time to the most recent common ancestor (tMRCA) for the tree root was 460 years (95% HPD 242–755 years). The tMRCAs estimated for the European clades (V and VI) indicate a relatively recent emergence. Thus, the clade V (Europe 1) had a mean tMRCA of 82 years, whereas the clade VI (Europe 2) had a mean tMRCA of 115 years.

## Discussion

CCHFV is nowadays considered the most widely distributed tick-borne virus in the world. However, it is restricted to the sub-tropical and tropical regions of the Old World and here apparently closely linked to the presence of *Hyalomma* spp. ticks as its main vector and reservoir [1]. Heneberg et al. [10] were the first to report *H. marginatum* (sensu syn. *H. plumbeum plumbeum*) from Kosovo, and by then the species accounted for about half of the total tick population on domestic animals. However, Oswald [11], in former southern Yugoslavia, including Kosovo, already noted a high incidence of what he then called *H. scupense*, which could well have been *H. marginatum*, considering the long-existing taxonomic confusion in the genus *Hyalomma*. Only now is it possible to compare our findings with other more recent studies on the Balkans and Turkey. While we found that about 57% of all ticks belonged to *H. marginatum*, the species accounts for 90% in the CCHF-endemic regions of Kosovo, figures that would seem to confirm its role as the main vector. Table S2 provides a review of literature on *H. marginatum* proportions in relation to CCHF endemicity. In the CCHF endemic foci in the Balkans and Turkey, *H. marginatum* comprise 10 to 98% of the total tick population (average 56%), whereas the proportion in non-endemic areas does not exceed 24% (mean 11%). However, this does not take into account micro-habitat and temporal differences. Nor does it consider the presence or absence of other *Hyalomma* species on the one hand, and the influence of potential vector species other than of the genus *Hyalomma* on the other. The present results only reflect the situation during the 2012 season. In a pilot study in May 2010 (data not shown) we found that 10% of all ticks belonged to *R. bursa* in Malishevë municipality, compared to zero in 2012. CCHFV has been isolated from more than 30 ixodid species [30], [31], but the genus *Hyalomma* and *H. marginatum* in particular are still considered the main vectors. Our results confirm this status for Kosovo, where 29 out of 40 positive ticks (72.5%) belonged to *H. marginatum*. During the 2001 outbreak all PCR-CCHFV-positive ticks were *H. marginatum* [5], [14].

Our phylogenetic analysis supports previous studies that reported the genetic heterogeneity of CCHFV worldwide and found that the existing isolates can be classified into seven main geographical clades: three African, two Asian and two European [32], [33]. Kosovar CCHFV sequences derived during this study fell within clades V and VI and cluster into two lineages designated as Kosovo I and IIb. These lineages are defined to a great extent on a temporal basis. Kosovo I lineage strains belonging to the largely divergent clade VI (Europe 2) seem to be carried exclusively by *R. bursa*. Clade VI also contains strains isolated from ticks in Greece (including the prototype strain AP92) and from humans in Turkey. These strains are currently associated with sub-clinical or mild cases [4], [34]. Kosovo IIa (human-derived) and IIb (tick-derived) lineages belong to a widespread clade of CCHFV (Europe 1) variants perpetuated mainly by *H. marginatum*. This suggests that the Kosovo outbreaks most likely originate from *H. marginatum* derived CCHFV. The existence of distinct lineages in Kosovo reflects the fact that CCHFV circulates in multiple areas that are separated from each other by geographic barriers such as climate, vegetation, and hosts. Thus, the adaptation of CCHFV to region-specific vectors and hosts leads to the emergence of local virus variants with different pathogenicity for humans. To determine whether viruses with different levels of virulence circulate within Kosovo, further clinical studies are required, including mild and subclinical cases. However, contrary to a previous report [7], our results suggest a spatial structure of the genetic variability of CCHFV in Kosovo. Thus, tick-derived CCHFV strains from Kosovo IIb lineage, which together with human-derived Kosovo IIa lineage formed a distinct subclade within clade VI (Europe 2), were detected predominantly in the hyper-endemic regions. In contrast, the tick-derived Kosovo I lineage strains (AP92-like) from clade V (Europe 1) were detected exclusively in *R. bursa* ticks in a non-endemic region, i.e. the outskirts of the capital Prishtinë (Fig. 1). Until recently, the AP92 strain, which was recovered from *R. bursa* ticks in Greece in 1978 [35], was thought to be avirulent for humans. In fact, the first symptomatic case in Greece was reported in 2008, but turned out to belong to clade VI [36]. However, recent studies reported AP92-like strains from patients with mild CCHF in the European part of Turkey, highlighting the potential pathogenicity for humans [34], [37], [38]. The source of infection was not clear, but local surveys of tick species revealed *Hyalomma* species and *R. bursa* as the main vectors for this variant. Contrary to these results, the Kosovar AP92-like variant seems to be maintained exclusively by *R. bursa* ticks.

Our phylogeographic reconstruction suggests that all seven CCHFV clades shared a common ancestor that existed about 500 years ago, most probably in Africa. This narrows down estimates from previous results, which calculated the existence of a common ancestor between 700 and 1000 years [7], [39]. The differences may be due to the large number of strains (n = 270) used in our study, resulting in a more robust time-resolved phylogenetic estimation. The phylogeographic reconstruction revealed two distinct successive introductions of CCHFV in Kosovo: the most probable location of the MRCA of the Kosovo I lineage (clade VI) was in Greece in 1949, and for lineage IIa and IIb in Turkey in 1977. Our results support previous studies, which reported a complex web of viral introductions/transmissions from Turkey to Kosovo [39]. Furthermore, our data also support the thesis that Europe experienced at least two distinct introductions of two highly divergent CCHFV strains, the first from Africa in the 1800s (low or apathogenic virus present in vector and animal host populations from Greece and Turkey), and the second in the first decades of the 1900s, when a more pathogenic strain spread, a strain that to this day is responsible for human outbreaks in eastern European countries [39]. In order to calculate the tMRCAs for the root and internal nodes of the clades V and VI, we estimated a mean evolutionary rate of 5.71×10^−4^ for all of the sampled CCHFV variant clades, 1.71×10^−4^ for clade V and 2.45×10^−3^ for clade VI. These estimates are higher than previously reported [39], [40], and suggest a rapid evolution of CCHFV. This is in concordance with the estimated evolutionary rates of the majority of other ssRNA viruses. However, the evolution of CCHFV is driven by two factors: firstly by the maintenance of high viral fitness due to alternating arthropod and vertebrate hosts' environments, and secondly, in all probability, by high polymerase error rates as observed in negative stranded RNA viruses [4], [41]. Moreover, given that the CCHFV is a segmented virus, the re-assorting/recombination events could be considered a major force driving its evolution [33]. However, in our dataset no consensus for recombination events was found using recombination detection methods.

Overall, the study provides the first insight into the genetic variability of CCHFV in tick populations from Kosovo. Ancestral reconstruction of CCHFV in Kosovar tick populations reveals two distinct CCHFV genotypes, which were introduced from Greece and Turkey and have been present in Kosovo for a long time. Our findings indicate that the virus is also spreading to non-endemic areas, which highlights the importance of further investigations involving both continuous surveillance and further genetic characterization of the CCHFV in the tick populations from hyper-endemic and non-endemic regions, which may be very important for monitoring and predicting future CCHF outbreaks in Kosovo.

## Supporting Information

Table S1 Details of all sequences used in the study including accession numbers, dates of sample collection, host species and countries of origin.
(DOC)

Table S2 Literature review on *H. marginatum* proportions in relation to CCHF endemicity.
(DOCX)
