# Supplementary material for: Crimean-Congo Hemorrhagic Fever Virus Clades V and VI (Europe 1 and 2) in Ticks in Kosovo, 2012
Source: PLoS Negl Trop Dis. 2014 Sep 25;8(9):e3168. doi: 10.1371/journal.pntd.0003168 (PMC4177860; doi:10.1371/journal.pntd.0003168)
Supplement: Table S1 — Details of all sequences used in the study including accession numbers, dates of sample collection, host species and countries of origin. (DOC) [file pntd.0003168.s001.doc]

Supplementary Table S1.

| Accession number | Country of origin | Host/Vector | Year of isolation |
| --- | --- | --- | --- |
|  |  |  |  |
| JX908640 | Afghanistan | *Homo sapiens* | 2012 |
| AF449482 | Albania | *Homo sapiens* | 2001 |
| KC846093 | Albania | *Homo sapiens* | 2003 |
| KC846094 | Albania | *Homo sapiens* | 2004 |
| AY277676 | Bulgaria | *Homo sapiens* | 1978 |
| GU477489 | Bulgaria | *Homo sapiens* | 1981 |
| AY550258 | Bulgaria | *Homo sapiens* | 2002 |
| AY550253 | Bulgaria | *Homo sapiens* | 2002 |
| AY550254 | Bulgaria | *Homo sapiens* | 2002 |
| AY550255 | Bulgaria | *Homo sapiens* | 2002 |
| AY550256 | Bulgaria | *Homo sapiens* | 2002 |
| JF807435 | Bulgaria | *Homo sapiens* | 2005 |
| JF807433 | Bulgaria | *Homo sapiens* | 2006 |
| FJ445749 | Bulgaria | *Homo sapiens* | 2008 |
| FJ472634 | Bulgaria | *Homo sapiens* | 2008 |
| FJ160262 | Bulgaria | *Homo sapiens* | 2008 |
| JF807428 | Bulgaria | *Homo sapiens* | 2009 |
| GU138618 | Bulgaria | *Homo sapiens* | 2009 |
| CHU15093 | Burkina Faso | *Homo sapiens* | 1983 |
| CHU15092 | Central African Republic | *Homo sapiens* | 1973 |
| GU477494 | China | *Euchoreutes naso* | 1979 |
| AJ010648 | China | *Homo sapiens* | 1965 |
| EU715258 | China | *Homo sapiens* | 1965 |
| EU715259 | China | *Homo sapiens* | 1974 |
| AF362080 | China | *Homo sapiens* | 1975 |
| AF354296 | China | *Homo sapiens* | 1978 |
| EU15261 | China | *Homo sapiens* | 1980 |
| AY029157 | China | *Homo sapiens* | 1988 |
| AB102853 | China | *Homo sapiens* | 2002 |
| AB209948 | China | *Homo sapiens* | 2002 |
| AB209945 | China | *Homo sapiens* | 2002 |
| AB209951 | China | *Hyalomma asiaticum* | 2004 |
| EU715278 | China | *Ovis aries* | 2005 |
| EU715268 | China | *Rattus rattus* | 2004 |
| EU715257 | China | Tick/no information | 1966 |
| CHU88413 | China | Tick/no information | 1968 |
| EU715262 | China | Tick/no information | 1980 |
| AJ010649 | China | Tick/no information | 1984 |
| EU715263 | China | Tick/no information | 1990 |
| EU715273 | China | Tick/no information | 2004 |
| EU715274 | China | Tick/no information | 2004 |
| EU715281 | China | Tick/no information | 2005 |
| EU715277 | China | Tick/no information | 2005 |
| EU715280 | China | Tick/no information | 2005 |
| DQ211650 | Congo | *Homo sapiens* | 1956 |
| HQ849545 | Congo | *Homo sapiens* | 2008 |
| EU871766 | Greece | *Homo sapiens* | 2008 |
| DQ211638 | Greece | *Rhipicephalus bursa* | 1975 |
| KF146306 | Greece | *Rhipicephalus bursa* | 2012 |
| JX051650 | India | *Hyalomma spp* | 2011 |
| AJ538196 | Irak | *Homo sapiens* | 1979 |
| CHU15022 | Iran | *Homo sapiens* | 1978 |
| AY905654 | Iran | *Homo sapiens* | 2000 |
| AY905655 | Iran | *Homo sapiens* | 2000 |
| AY366377 | Iran | *Homo sapiens* | 2002 |
| AY366375 | Iran | *Homo sapiens* | 2002 |
| GU456727 | Iran | *Hyalomma anatolicum* | 2007 |
| GU456723 | Iran | *Rhipicephalus sanguineus* | 2007 |
| JF798866 | Iran | Tick/no information | 2010 |
| AF362743 | Kazakhstan | *Homo sapiens* | 2000 |
| JN173797 | Kosovo | *Homo sapiens* | 2001 |
| DQ133507 | Kosovo | *Homo sapiens* | 2001 |
| AF428145 | Kosovo | *Homo sapiens* | 2001 |
| KC477805 | Kosovo | *Homo sapiens* | 2012 |
| KC477811 | Kosovo | *Homo sapiens* | 2012 |
| KC477787 | Kosovo | *Homo sapiens* | 2012 |
| KC477782 | Kosovo | *Homo sapiens* | 2012 |
| KC477827 | Kosovo | *Homo sapiens* | 2012 |
| KC477830 | Kosovo | *Homo sapiens* | 2012 |
| KC477822 | Kosovo | *Homo sapiens* | 2012 |
| KC477823 | Kosovo | *Homo sapiens* | 2012 |
| KC477780 | Kosovo | *Homo sapiens* | 2012 |
| KC477783 | Kosovo | *Homo sapiens* | 2012 |
| KC477824 | Kosovo | *Homo sapiens* | 2012 |
| KC477789 | Kosovo | *Homo sapiens* | 2012 |
| KC477805 | Kosovo | *Homo sapiens* | 2012 |
| KC477819 | Kosovo | *Homo sapiens* | 2012 |
| KJ545674 | Kosovo | *Hyalomma marginatum* | 2012 |
| KJ545672 | Kosovo | *Hyalomma marginatum* | 2012 |
| KJ545679 | Kosovo | *Hyalomma marginatum* | 2012 |
| KJ545680 | Kosovo | *Hyalomma marginatum* | 2012 |
| KJ545684 | Kosovo | *Hyalomma marginatum* | 2012 |
| KJ545694 | Kosovo | *Hyalomma marginatum* | 2012 |
| KJ545683 | Kosovo | *Hyalomma marginatum* | 2012 |
| KJ545689 | Kosovo | *Hyalomma marginatum* | 2012 |
| KJ545676 | Kosovo | *Hyalomma marginatum* | 2012 |
| KJ545668 | Kosovo | *Hyalomma marginatum* | 2012 |
| KJ545666 | Kosovo | *Hyalomma marginatum* | 2012 |
| KJ545665 | Kosovo | *Hyalomma marginatum* | 2012 |
| KJ545667 | Kosovo | *Hyalomma marginatum* | 2012 |
| KJ545688 | Kosovo | *Hyalomma marginatum* | 2012 |
| KJ545675 | Kosovo | *Hyalomma marginatum* | 2012 |
| KJ545691 | Kosovo | *Hyalomma marginatum* | 2012 |
| KJ545671 | Kosovo | *Hyalomma marginatum* | 2012 |
| KJ545673 | Kosovo | *Hyalomma marginatum* | 2012 |
| KJ545682 | Kosovo | *Hyalomma marginatum* | 2012 |
| KJ545681 | Kosovo | *Hyalomma marginatum* | 2012 |
| KJ545677 | Kosovo | *Hyalomma marginatum* | 2012 |
| KJ545686 | Kosovo | *Hyalomma marginatum* | 2012 |
| KJ545690 | Kosovo | *Hyalomma marginatum* | 2012 |
| KJ545687 | Kosovo | *Hyalomma marginatum* | 2012 |
| KJ545685 | Kosovo | *Hyalomma marginatum* | 2012 |
| KJ545670 | Kosovo | *Hyalomma marginatum* | 2012 |
| KJ545678 | Kosovo | *Hyalomma marginatum* | 2012 |
| KJ545693 | Kosovo | *Hyalomma marginatum* | 2012 |
| KJ545669 | Kosovo | *Hyalomma marginatum* | 2012 |
| KJ545692 | Kosovo | *Ixodes ricinus* | 2012 |
| KJ545704 | Kosovo | *Rhipicephalus bursa* | 2012 |
| KJ545701 | Kosovo | *Rhipicephalus bursa* | 2012 |
| KJ545699 | Kosovo | *Rhipicephalus bursa* | 2012 |
| KJ545696 | Kosovo | *Rhipicephalus bursa* | 2012 |
| KJ545695 | Kosovo | *Rhipicephalus bursa* | 2012 |
| KJ545703 | Kosovo | *Rhipicephalus bursa* | 2012 |
| KJ545697 | Kosovo | *Rhipicephalus bursa* | 2012 |
| KJ545702 | Kosovo | *Rhipicephalus bursa* | 2012 |
| KJ545698 | Kosovo | *Rhipicephalus bursa* | 2012 |
| KJ545700 | Kosovo | *Rhipicephalus bursa* | 2012 |
| CHU15024 | Madagascar | *Homo sapiens* | 1985 |
| CHU15089 | Mauritania | *Homo sapiens* | 1984 |
| CHU15023 | Mauritania | *Homo sapiens* | 1988 |
| DQ211641 | Mauritania | *Hyalomma rufipes* | 1984 |
| AY905630 | Namibia | *Homo sapiens* | 1986 |
| AY905632 | Namibia | *Homo sapiens* | 1986 |
| FJ435413 | Namibia | *Homo sapiens* | 1988 |
| FJ435419 | Namibia | *Homo sapiens* | 1988 |
| AY905640 | Namibia | *Homo sapiens* | 1998 |
| AY905648 | Namibia | *Homo sapiens* | 2001 |
| AY905652 | Namibia | *Homo sapiens* | 2001 |
| DQ211645 | Oman | *Homo sapiens* | 1997 |
| CHU88414 | Pakistan | *Homo sapiens* | 1965 |
| AF527810 | Pakistan | *Homo sapiens* | 1976 |
| CHU75677 | Pakistan | *Homo sapiens* | 1976 |
| KC869991 | Pakistan | *Homo sapiens* | 2002 |
| KC869989 | Pakistan | *Homo sapiens* | 2008 |
| DQ211643 | Russia | *Homo sapiens* | 1967 |
| DQ211644 | Russia | *Homo sapiens* | 1967 |
| AF481802 | Russia | *Homo sapiens* | 2000 |
| DQ206447 | Russia | *Homo sapiens* | 2002 |
| KF494375 | Russia | *Homo sapiens* | 2011 |
| DQ211640 | Senegal | *Capra aegagrus hircus* | 1972 |
| CHU15020 | Senegal | *Homo sapiens* | 1973 |
| CHU15090 | Senegal | *Homo sapiens* | 1993 |
| CHU15091 | Senegal | *Homo sapiens* | 1993 |
| DQ211639 | Senegal | *Hyalomma truncatum* | 1969 |
| DQ076416 | South Africa | *Homo sapiens* | 1981 |
| AY905625 | South Africa | *Homo sapiens* | 1984 |
| AY905626 | South Africa | *Homo sapiens* | 1985 |
| CHU84636 | South Africa | *Homo sapiens* | 1985 |
| AY905627 | South Africa | *Homo sapiens* | 1985 |
| DQ211646 | South Africa | *Homo sapiens* | 1985 |
| DQ211648 | South Africa | *Homo sapiens* | 1985 |
| CHU84635 | South Africa | *Homo sapiens* | 1986 |
| AY905633 | South Africa | *Homo sapiens* | 1986 |
| AY905631 | South Africa | *Homo sapiens* | 1986 |
| AY905628 | South Africa | *Homo sapiens* | 1986 |
| CHU84638 | South Africa | *Homo sapiens* | 1986 |
| AY905629 | South Africa | *Homo sapiens* | 1986 |
| CHU84639 | South Africa | *Homo sapiens* | 1987 |
| DQ211647 | South Africa | *Homo sapiens* | 1987 |
| FJ435416 | South Africa | *Homo sapiens* | 1987 |
| AY905634 | South Africa | *Homo sapiens* | 1987 |
| FJ435420 | South Africa | *Homo sapiens* | 1988 |
| FJ435412 | South Africa | *Homo sapiens* | 1988 |
| CHU84637 | South Africa | *Homo sapiens* | 1988 |
| AY905635 | South Africa | *Homo sapiens* | 1988 |
| FJ435423 | South Africa | *Homo sapiens* | 1988 |
| AY905637 | South Africa | *Homo sapiens* | 1989 |
| FJ435421 | South Africa | *Homo sapiens* | 1989 |
| FJ435414 | South Africa | *Homo sapiens* | 1989 |
| FJ435417 | South Africa | *Homo sapiens* | 1989 |
| AY905636 | South Africa | *Homo sapiens* | 1989 |
| FJ435415 | South Africa | *Homo sapiens* | 1990 |
| AY905638 | South Africa | *Homo sapiens* | 1990 |
| AY905639 | South Africa | *Homo sapiens* | 1998 |
| AY905641 | South Africa | *Homo sapiens* | 1999 |
| AY905642 | South Africa | *Homo sapiens* | 1999 |
| AY905644 | South Africa | *Homo sapiens* | 2000 |
| AY905647 | South Africa | *Homo sapiens* | 2000 |
| AY905645 | South Africa | *Homo sapiens* | 2000 |
| AY905646 | South Africa | *Homo sapiens* | 2000 |
| AY905643 | South Africa | *Homo sapiens* | 2000 |
| AY905651 | South Africa | *Homo sapiens* | 2001 |
| KC880335 | South Africa | *Homo sapiens* | 2001 |
| AY905650 | South Africa | *Homo sapiens* | 2001 |
| AY905649 | South Africa | *Homo sapiens* | 2001 |
| GQ862371 | Sudan | *Homo sapiens* | 2008 |
| HQ378179 | Sudan | *Homo sapiens* | 2009 |
| HQ829854 | Sudan | *Homo sapiens* | 2010 |
| KF725870 | Syria | *Hyalomma aegyptium* | 2007 |
| AY297692 | Tajikistan | *Homo sapiens* | 1990 |
| AF481805 | Tajikistan | *Homo sapiens* | 1990 |
| AY049083 | Tajikistan | *Homo sapiens* | 1990 |
| AY297691 | Tajikistan | *Homo sapiens* | 1991 |
| JN086996 | Tajikistan | *Homo sapiens* | 2010 |
| AY045567 | Tajikistan | *Hyalomma marginatum* | 2000 |
| GU324994 | Turkey | *Homo sapiens* | 1999 |
| EF432640 | Turkey | *Homo sapiens* | 2004 |
| EF432639 | Turkey | *Homo sapiens* | 2004 |
| EF432653 | Turkey | *Homo sapiens* | 2004 |
| DQ397823 | Turkey | *Homo sapiens* | 2005 |
| DQ397825 | Turkey | *Homo sapiens* | 2005 |
| DQ397824 | Turkey | *Homo sapiens* | 2005 |
| EF432647 | Turkey | *Homo sapiens* | 2005 |
| DQ397826 | Turkey | *Homo sapiens* | 2005 |
| FJ601866 | Turkey | *Homo sapiens* | 2006 |
| DQ983742 | Turkey | *Homo sapiens* | 2006 |
| GQ337053 | Turkey | *Homo sapiens* | 2006 |
| DQ983745 | Turkey | *Homo sapiens* | 2006 |
| DQ983743 | Turkey | *Homo sapiens* | 2006 |
| FJ601876 | Turkey | *Homo sapiens* | 2007 |
| EU727456 | Turkey | *Homo sapiens* | 2007 |
| FJ601875 | Turkey | *Homo sapiens* | 2007 |
| FJ601877 | Turkey | *Homo sapiens* | 2007 |
| FJ601874 | Turkey | *Homo sapiens* | 2007 |
| FJ601870 | Turkey | *Homo sapiens* | 2007 |
| EU057975 | Turkey | *Homo sapiens* | 2007 |
| FJ601872 | Turkey | *Homo sapiens* | 2007 |
| FJ601858 | Turkey | *Homo sapiens* | 2008 |
| FJ601847 | Turkey | *Homo sapiens* | 2008 |
| FJ601852 | Turkey | *Homo sapiens* | 2008 |
| FJ601890 | Turkey | *Homo sapiens* | 2008 |
| FJ601893 | Turkey | *Homo sapiens* | 2008 |
| FJ601882 | Turkey | *Homo sapiens* | 2008 |
| FJ601896 | Turkey | *Homo sapiens* | 2008 |
| FJ601845 | Turkey | *Homo sapiens* | 2008 |
| FJ601853 | Turkey | *Homo sapiens* | 2008 |
| FJ601851 | Turkey | *Homo sapiens* | 2008 |
| GU324989 | Turkey | *Homo sapiens* | 2008 |
| FJ392604 | Turkey | *Homo sapiens* | 2008 |
| HQ188922 | Turkey | *Homo sapiens* | 2009 |
| GU084162 | Turkey | *Homo sapiens* | 2009 |
| HQ685840 | Turkey | *Homo sapiens* | 2009 |
| HQ188915 | Turkey | *Homo sapiens* | 2009 |
| HQ166288 | Turkey | *Homo sapiens* | 2009 |
| HQ821873 | Turkey | *Homo sapiens* | 2009 |
| HQ829812 | Turkey | *Homo sapiens* | 2009 |
| HQ188914 | Turkey | *Homo sapiens* | 2009 |
| HQ173898 | Turkey | *Homo sapiens* | 2009 |
| HQ173900 | Turkey | *Homo sapiens* | 2009 |
| HQ173897 | Turkey | *Homo sapiens* | 2009 |
| HQ829817 | Turkey | *Homo sapiens* | 2009 |
| HQ675002 | Turkey | *Homo sapiens* | 2010 |
| HQ664913 | Turkey | *Homo sapiens* | 2010 |
| HQ829819 | Turkey | *Homo sapiens* | 2010 |
| HQ829809 | Turkey | *Homo sapiens* | 2010 |
| HQ685839 | Turkey | *Homo sapiens* | 2010 |
| HQ833035 | Turkey | *Homo sapiens* | 2010 |
| HQ188918 | Turkey | *Homo sapiens* | 2010 |
| HQ821875 | Turkey | *Homo sapiens* | 2010 |
| HQ188920 | Turkey | *Homo sapiens* | 2010 |
| HQ829810 | Turkey | *Homo sapiens* | 2010 |
| HQ188921 | Turkey | *Homo sapiens* | 2010 |
| KF725872 | Turkey | *Hyalomma aegyptium* | 2007 |
| EF012361 | Turkey | *Hyalomma marginatum* | 2005 |
| EF012362 | Turkey | *Hyalomma marginatum* | 2005 |
| DQ983785 | Turkey | *Hyalomma marginatum* | 2006 |
| FJ392601 | Turkey | *Rhipicephalus bursa* | 2008 |
| FJ392603 | Turkey | *Rhipicephalus bursa* | 2008 |
| KF727977 | Turkey | Tick/no information | 2010 |
| KF727976 | Turkey | Tick/no information | 2010 |
| CHU88416 | Uganda | *Homo sapiens* | 1956 |
| NC005302 | Uganda | *Homo sapiens* | 1966 |
| JN108025 | United Arab Emirates | *Homo sapiens* | 1979 |
| S82581 | United Arab Emirates | *Homo sapiens* | 1993 |
| CHU75670 | United Arab Emirates | *Homo sapiens* | 1994 |
| CHU75672 | United Arab Emirates | *Homo sapiens* | 1995 |
| CHU75668 | United Arab Emirates | *Homo sapiens* | 1995 |
| CHU75669 | United Arab Emirates | *Hyalomma anatolicum* | 1995 |
| CHU75673 | United Arab Emirates | *Hyalomma impeltatum* | 1995 |
| AY223475 | Uzbekistan | *Homo sapiens* | 1967 |
| AF481799 | Uzbekistan | Tick/no information | 1985 |
