# Supplementary material for: Crimean-Congo Hemorrhagic Fever Virus Clades V and VI (Europe 1 and 2) in Ticks in Kosovo, 2012
Source: PLoS Negl Trop Dis. 2014 Sep 25;8(9):e3168. doi: 10.1371/journal.pntd.0003168 (PMC4177860; doi:10.1371/journal.pntd.0003168)
Supplement: Table S2 — Literature review on H. marginatum proportions in relation to CCHF endemicity. (DOCX) [file pntd.0003168.s002.docx]

Supplementary Table S2.

| **Region** | **Host,**  **collected from** | **Proportion (%) of *H. marginatum* in total tick population** | **CCHF endemicity** | **Reference** |
| --- | --- | --- | --- | --- |
| Kosovo, central basin | Livestock | 90 | Hyper | Present study |
| Kosovo | Cattle | 98 | Hyper | [5] Avšič-Županc (2007) |
| FYROM, Čiflik | Livestock | 48-74 | Endemic | Gligić et al. (1980) |
| Bulgaria, central-southeastern | Livestock | 31 | Endemic | Gergova et al. (2012) |
| Albania, norteastern | Cattle | 10-40 | Endemic | [3] Papa et al. (2009) |
| Turkey, central-north | Livestock, human | 18-46  (60-64% *H. spp.*) | Endemic | Bursali et al. (2011), [31] Tekin et al. (2012), Tonbak et al. (2006) |
| Kosovo, peripheral foothills | Livestock, ground | 24 | No/low | Present study |
| Serbia, central | Livestock, dogs | 0 | No | Milutinović & Radulović (2002) |
| Bosnia & Herzegovina | Livestock, dogs, rodents, human, vegetation | 5.7 | No | Omeragic (2011) |
| Greece, northern | Livestock  Human | 12.4  5.2 | Low | Pavlidou et al.(2008)  Papa et al. (2011) |
| Turkey, Ankara | Livestock | 5.4 | Low | Hekimoglu et al. (2012) |
| Turkey, South Marmara | Livestock | 15  (30% *H. spp.*) | No/low | Yesilbag et al. (2013) |
| Turkey, Thrace | Cattle | *ca*. 24 | No/low | Gargili et al. (2011) |

References

Bursali A, Tekin S, Keskin A, Ekici M, Dundar E (2011) Species diversity of ixodid ticks feeding on humans in Amasya, Turkey: seasonal abundance and presence of Crimean-Congo hemorrhagic fever virus. J Med Entomol 48: 85-93.

Gargili A, Midilli K, Ergonul O, Ergin S, Alp HG, et al. (2011) Crimean-Congo hemorrhagic fever in European part of Turkey: genetic analysis of the virus strains from ticks and a seroepidemiological study in humans. Vector Borne Zoonotic Dis 11: 747-752.

Gergova I, Kunchev M, Kamarinchev B (2012) Crimean-Congo hemorrhagic fever virus – tick survey in endemic areas in Bulgaria. J Med Viro 84: 608-614.

Gligić A, Stojanović R, Obradović M, Bošković R (1980) Serological examination of Crimean-Congo hemorrhagic fever infections of domestic animals in natural foci. Zbl Bakt Suppl 9: 263-266.

Hekimoglu O, Ozer N, Ergunay K, Ozkul A (2012) Species distribution and detection of Crimean Congo Hemorrhagic Fever Virus (CCHFV) in field-collected ticks in Ankara Province, Central Anatolia, Turkey. Exp Appl Acarol 56: 75-84.

Milutinović M, Radulović Z (2002) Ecological notes on ticks (Acari: Ixodidae) in Serbia (central regions). Acta Veterinaria (Beograd) 52: 49-58.

Omeragic J (2011) Ixodid ticks in Bosnia and Herzegovina. Exp Appl Acarol 53: 301-309.

Papa A, Chaligiannis I, Xanthopoulou K, Papaioakim M, Papanastasiou S, et al. (2011) Ticks parasitizing humans in Greece. Vector Borne Zoonotic Dis 11: 539-542.

Pavlidou V, Gerou S, Kahrimanidou M, Papa A (2008) Ticks infesting domestic animals in northern Greece. Exp Appl Acarol 45: 195-198.

Tonbak S, Aktas M, Altay K, Azkur AK, Kalkan A, et al. (2006) Crimean-Congo hemorrhagic fever virus: genetic analysis and tick survey in Turkey. J Clin Microbiol 44: 4120-4124.

Yesilbag K, Aydin L, Dincer E, Alpay G, Girisgin AO, et al. (2013) Tick survey and detection of Crimean-Congo hemorrhagic fever virus in tick species from a non-endemic area, South Marmara region, Turkey. Exp Appl Acarol 60: 253-261.
